# Burning fat with cysteine depletion

**DOI:** 10.15698/cst2025.11.313

**Published:** 2025-11-13

**Authors:** Brittney Adams, Stanislaw Walkowiak, Mohammed K. Hankir

**Affiliations:** 1 School of Biochemistry and Immunology, Trinity Biomedical Sciences Institute, Trinity College Dublin, Dublin, Ireland.

**Keywords:** essential amino acids, cysteine, integrated stress response, oxidative stress response, adipose tissue thermogenesis, obesity

## Abstract

Removing certain essential amino acids from the diet is known to promote weight loss in rodents via effects on food intake and energy expenditure. Two complementary articles by Varghese et al [Nature 643(8072)] and Lee et al [Nature Metabolism 7(6)] now show that cysteine depletion through combined dietary and genetic means in mice evokes a unique stress response in the liver to amplify these metabolic outcomes and offer a potentially new treatment option for obesity.

## Burning fat with cysteine depletion

Obesity is an ever-growing health and socioeconomic crisis 1,2,3,4. When attempting to understand the global surge in obesity rates over the last half century, changes in the food system that have made our diets more accessible, processed, palatable and energy dense than ever before have emerged as a major driver 5. In efforts to combat obesity various treatments have been developed including lifestyle modifications such as dieting and exercise, drugs such as the glucagon-like peptide 1 (GLP-1) receptor agonist semaglutide, and bariatric surgeries such as Roux-en-Y gastric bypass (RYGB) 6. Although beneficial for many, they have failed to revert the projected upward trajectory of global obesity rates 7, underscoring the need for alternative approaches.

High-protein diets are well known to promote weight loss in the clinical setting due to their ability to enhance the release of satiety hormones from the gut like GLP-1 as well as the considerably greater energy it requires for protein digestion compared to other macronutrients 8. Perhaps counterintuitively then, diets missing individual essential amino acids have similar weight lowering effects in preclinical studies, although the underlying mechanisms differ 9. For example, leucine deprivation promotes weight loss by aversively reducing food intake and stimulating brown adipose tissue thermogenesis 10. This was shown to be mediated in neurons of the anterior piriform cortex by the uncharged tRNA sensor and enzyme kinase general control nonderepressible 2 (GCN2) 11,12, a core component of the integrated stress response (ISR) that causes cessation of protein translation through the phosphorylation of eukaryotic initiation factor 2-α (eIF2-α) 13,14. On the other hand, despite increasing food intake, methionine deprivation promotes weight loss through the browning of retroperitoneal adipose tissue 15 - an often overlooked fat depot where thermogenic gene expression strongly associates with body weight in mice 16 - although this appears to be independent of GCN2 signalling 17. Notably, methionine deprivation also reduces systemic cysteine levels, and its supplementation under these conditions normalizes body weight 18. While cysteine is not an essential amino acid, it is a component of vital molecules involved in cellular metabolism such as the antioxidant glutathione (GSH) and coenzyme A (CoA). Despite its obvious importance, the independent role of cysteine in regulating whole-body energy homeostasis remains unclear. Varghese et al 1 and Lee et al 2 now use distinct yet complementary approaches to comprehensively address this question in mice.

In the first study, Varghese et al. 1 systematically tested deprivation of all nine essential amino acids as well as cysteine on body weight in mice lacking cystathionine-gamma-lyase (CSE), one of two enzymes involved in cysteine production from homocysteine, and heterozygous controls. It should be noted that the employed CSE knockout mouse model has questionable deletion of CSE in the liver based on the Western blot provided in the original publication 19.

Also, these mice were of a normal starting body weight (20-29 g), which weakens the potential applicability of the findings of Varghese et al. 1 for the treatment of obesity. These issues aside, deprivation of any of the essential amino acids from the diet for 7 days led to rapid weight loss in heterozygous mice ranging from approximately 10% (for lysine) to 25% (for threonine). On the other hand, cysteine deprivation was associated with approximately 5% body weight gain in these mice as for the control diet group, consistent with it being a non-essential amino acid. Strikingly, cysteine deprivation in CSE knockout mice (thus causing an acute state of cysteine depletion) led to a whopping 30% weight loss, exceeding that in any other group although isoleucine deprivation in this setting was not far behind (27%). Importantly, the effect of cysteine depletion on body weight could be fully reversed within days by simply reintroducing a standard cysteine replete diet.

Next, the authors determined the cause of the changes in body weight in CSE knockout mice deprived of cysteine. Similar to that described for leucine deprivation 10, cysteine depletion aversively reduced food intake possibly due to GCN2 signalling in the anterior piriform cortex 20, although this was not explored. Again, like leucine deprivation 10, a sizeable portion of weight loss (15%) was independent of food intake. Malabsorption was excluded based on metabolite measurements in faeces, although it would have been useful to perform the gold-standard bomb calorimetry experiments to measure total energy content. Gut microbiota were also excluded based on their depletion in CSE knockout mice deprived of cysteine with antibiotics. Indirect calorimetry revealed that respiratory exchange ratio (RER), which reflects fuel partitioning, progressively decreased upon cysteine depletion indicative of fatty acid utilisation over glucose while energy expenditure increased on day 3. Accordingly, immunohistochemistry revealed that subcutaneous adipose tissue underwent focal browning on day 3 as determined by increased uncoupling protein 1 (UCP1) expression, although curiously this subsided by day 7. Other forms of thermogenesis such as creatine cycling have been described in adipose tissue 21. Indeed, RNA sequencing revealed increased creatine kinase mRNA expression in epigonadal adipose tissue in CSE knockout mice after 2 days of cysteine deprivation although it would have been interesting to see how this was affected by day 7.

Varghese et al. 1 then explored the molecular mechanisms underlying the metabolic shift caused by cysteine depletion through RNA-sequencing of all major metabolic tissues (liver, muscle, and epigonadal adipose tissue). In the liver, they observed broad upregulation of genes linked to the ISR as well as genes associated with the nuclear factor erythroid 2-related factor (NRF2)-regulated oxidative stress response (OSR).

In epigonadal adipose tissue, no such stress response was noted although it would have been interesting to analyse subcutaneous adipose tissue considering this is where the browning effect was observed. This finding is nevertheless significant, as it provides the first clue that a potentially non-cell autonomous mechanism drives the remodelling of adipose tissue during cysteine depletion. In contrast, muscle tissue primarily showed activation of OSR genes, indicative of oxidative stress. The lack of ISR in muscle is consistent with the high amounts of protein in this tissue providing a local source of amino acids when they are no longer available in the diet as pointed out by the authors. In line with the RNA sequencing analysis showing an OSR in liver and muscle, GSH was reduced in these tissues but not in epigonadal adipose tissue. Together, these data suggest that cysteine depletion evokes a hitherto undescribed stress response in the liver: an ISR presumably due to the activation of GCN2 combined with an OSR due to local reductions in GSH. Unfortunately, the former could not be tested due to the lethality arising from combined deletion of GCN2 with CSE, although it would have been interesting to test the latter with combined deletion of NRF2 with CSE. These issues notwithstanding, to demonstrate the importance of the liver in the mediating the metabolic phenotype associated with cysteine depletion, an elegant rescue experiment was performed in which CSE was selectively re-expressed in this organ via adeno associated virus 8 (AAV8)-mediated gene transfer. Remarkably, this completely reversed the weight loss in CSE knockout mice resulting from cysteine deprivation and normalised GSH levels in the liver.

The data so far suggests that a secreted factor from the liver could mediate the effects of cysteine depletion on body weight. Growth and differentiation factor 15 (GDF15) and fibroblast growth factor 21 (FGF21) are two hepatokines that are increased under stressful conditions with established roles in regulating food intake and energy expenditure 22,23. Indeed, RNA sequencing revealed Gdf15 mRNA expression levels approximately doubled, while Fgf21 mRNA expression was strikingly induced in the liver during cysteine depletion. This pattern of gene expression was reflected in the circulating levels of GDF15 and FGF21 on day 3 and day 7. To test the requirement of GDF15 and FGF21 in the changes in body weight caused by cysteine depletion, CSE knockout mice were crossed with GDF15 and FGF21 knockout mice generating two lines of double knockouts. Somewhat unexpectedly, these mice exhibited similar weight loss to CSE knockout mice deprived of cysteine, although FGF21 deficiency did partially protect from weight loss. Considering potential compensation by one hepatokine for the other in both lines of double knockouts, it would be interesting to develop triple knockouts or at least to determine the effects of immunoneutralising GDF15 24 in the CSE and FGF21 double knockout mice.

Finally, the authors turned their attention to the other cysteine-dependent metabolite CoA. Similar to GSH, cysteine depletion led to a marked reduction in CoA levels in liver and a modest but significant reduction in muscle. Metabolomic analysis of urine samples revealed increases in metabolites associated with reactions preceding CoA-dependent steps in the tricarboxylic acid cycle. These findings suggest that under low levels of CoA, energy rich metabolites are lost through excretion in the urine possibly also contributing to weight loss. Because the essential vitamin pantothenic acid (vitamin B5) forms part of the CoA molecule, it would also be expected to contribute to the metabolic phenotype associated with cysteine depletion. Indeed, when CSE knockout mice were switched from a no cysteine to a no vitamin B5 diet, their body weights only partially recovered. This was associated with a partial recovery of CoA in the liver suggesting a preserved state of metabolic dysfunction although it would have been useful to perform metabolomic analysis of urine under these conditions to complete the picture.

In the second study, Lee et al. 2 started by investigating the metabolic impact of caloric restriction (CR) on subcutaneous adipose tissue in humans using samples from the CALERIE-II trial 25. After one year of CR with a 15% reduction in caloric intake and body weight, unbiased metabolomics revealed significant remodelling of cysteine, methionine, and taurine metabolism. Paradoxically, RNA sequencing revealed increased expression of CSE (termed CTH in this study) despite decreased cysteine and GSH levels in subcutaneous adipose tissue. Placing faith in the metabolomics data, the authors proceeded by generating their own CTH knockout mouse line to assess the metabolic impact of depleting cysteine in the body upon cystine deprivation in the diet. Similar to the findings of Varghese et al. 1, this led to 25-30% weight loss within one week which was fully reversible within 2 days by switching to a 75% cystine diet. Importantly, the authors demonstrated that cysteine levels in the circulation were indeed reduced by 66% in CTH knockout mice deprived of cysteine, while GSH levels were reduced in subcutaneous adipose tissue, brown adipose tissue and liver whereas CoA was reduced only in the latter two.

Next, Lee et al. 2 determined the cause of the profound weight loss associated with cysteine depletion. Unlike the findings of Varghese et al. 1, the aversive reduction in food intake did not extend beyond 2 days. Furthermore, bomb calorimetry of faecal matter was performed definitively revealing no effect on energy extraction from food. On the other hand, indirect calorimetry combined with ANCOVA analysis clearly revealed that energy expenditure was markedly increased along with a striking wholesale browning of subcutaneous adipose tissue and marked browning of epigonadal adipose tissue as determined by immunostaining for UCP1 on day 6. The authors did not stop there and performed detailed cell culture experiments on the stromal vascular fraction (SVF) of subcutaneous adipose tissue from CTH knockout mice. This elegantly revealed that cysteine-free media (unlike amino acid-restricted media) 26 does not induce the differentiation of preadipocytes into mature thermogenic adipocytes as determined by Ucp1 mRNA expression, proving non-cell autonomous mechanisms contribute to this effect. Indeed, single cell RNA sequencing of SVF revealed that a cluster of cells with markers of pre-adipocytes that respond to beta-3 adrenergic receptor agonists is enriched by cysteine depletion in line with enhanced sympathetic nervous system function. It should be stated at this point that these data are essentially at odds with the human metabolomics data suggesting reduced cysteine levels in subcutaneous adipose tissue drives the metabolic benefits of CR but nevertheless served as a useful starting point for the investigation. Interestingly, despite the increase in energy expenditure and extensive adipose tissue browning during cysteine depletion, core body temperature did not change under these conditions. A sensitive magnetic resonance spectroscopy technique previously used to measure brain temperature in rats called biosensor imaging of redundant deviation in shifts (BIRDS)^27^ did however reveal increased heat production in brown adipose tissue.

To determine the molecular mechanisms of weight loss in CTH knockout mice deprived of cysteine, Lee et al. 2 systematically developed no less than 2 lines of tissue specific CTH knockout mice and 3 lines of double knockout mice. Adipose tissue and liver specific CTH knockout mice were generated by crossing adiponectin-Cre and Lys-Cre mice with floxed CTH mice, respectively, but had normal body weight under conditions of cysteine deprivation. This is possibly because of redundancy in cysteine pathways since circulating cysteine was unaffected in these mice, unlike in global CTH knockouts. The authors then measured circulating GDF15 and FGF21 in CTH knockout mice during cysteine deprivation. This revealed a relatively modest but significant increase in GDF15 but a marked induction of FGF21 in the circulation by day 4. Similar to the findings of Varghese et al. 1, however, knockout of FGF21 only partially prevented the weight lowering effects of cysteine depletion. Because impaired homocysteine cycling in the liver leads to endoplasmic reticulum (ER) stress inducing FGF21 release 28, mice lacking the ER stress protein C/EBP homologous protein (CHOP) were crossed with CTH knockout mice. While this prevented the increase in circulating FGF21 during cysteine depletion it did not protect from weight loss. Finally, given the marked browning of adipose tissue and activation of brown adipose tissue in CTH knockout mice deprived of cysteine, the authors also generated double knockout mice lacking UCP1 although yet again no rescuing effect on weight loss was observed. These results suggest that creatine cycling may be more relevant in the context of cysteine depletion or even a novel form of futile cycling in adipose tissue as pointed out by the authors.

Adipose tissue thermogenesis is triggered by a thermoregulatory network in the brain culminating in noradrenaline release by sympathetic nerve endings 29. To test if a similar network is engaged by cysteine depletion, the authors performed whole-brain analysis of neuronal activation using immunolabeling-enabled three-dimensional imaging of solvent-cleared organs (iDISCO) 30. This revealed activation of sensory regions like the lateral parabrachial nucleus (PBN), processing regions like the hypothalamus and effector regions like the periaqueductal grey (PAG) and dorsal raphe nucleus (DRN). Accordingly, noradrenaline levels were increased in subcutaneous adipose tissue and brown adipose tissue which is also in line with the single-cell RNA sequencing data of SVF described above. Moreover, pharmacologically blocking beta-3 adrenergic receptor signalling with L748337 halved the weight loss that occurs during cysteine depletion associated with markedly reduced browning of subcutaneous adipose tissue as determined by Ucp1 mRNA levels.

Finally, to test the potential therapeutic relevance of cysteine depletion for treating obesity, it was applied to mildly diet-induced obese mice (body weights of 25-35g). Remarkably, these mice also exhibited a similar magnitude of weight loss to normal weight mice due to increased energy expenditure and browning of both subcutaneous and epigonadal adipose tissue. This was associated with approximately 50% reduction in fasting blood glucose and marked improvement in glucose tolerance suggesting cysteine depletion could be a useful treatment for hyperglycaemia.

**Figure 1:**
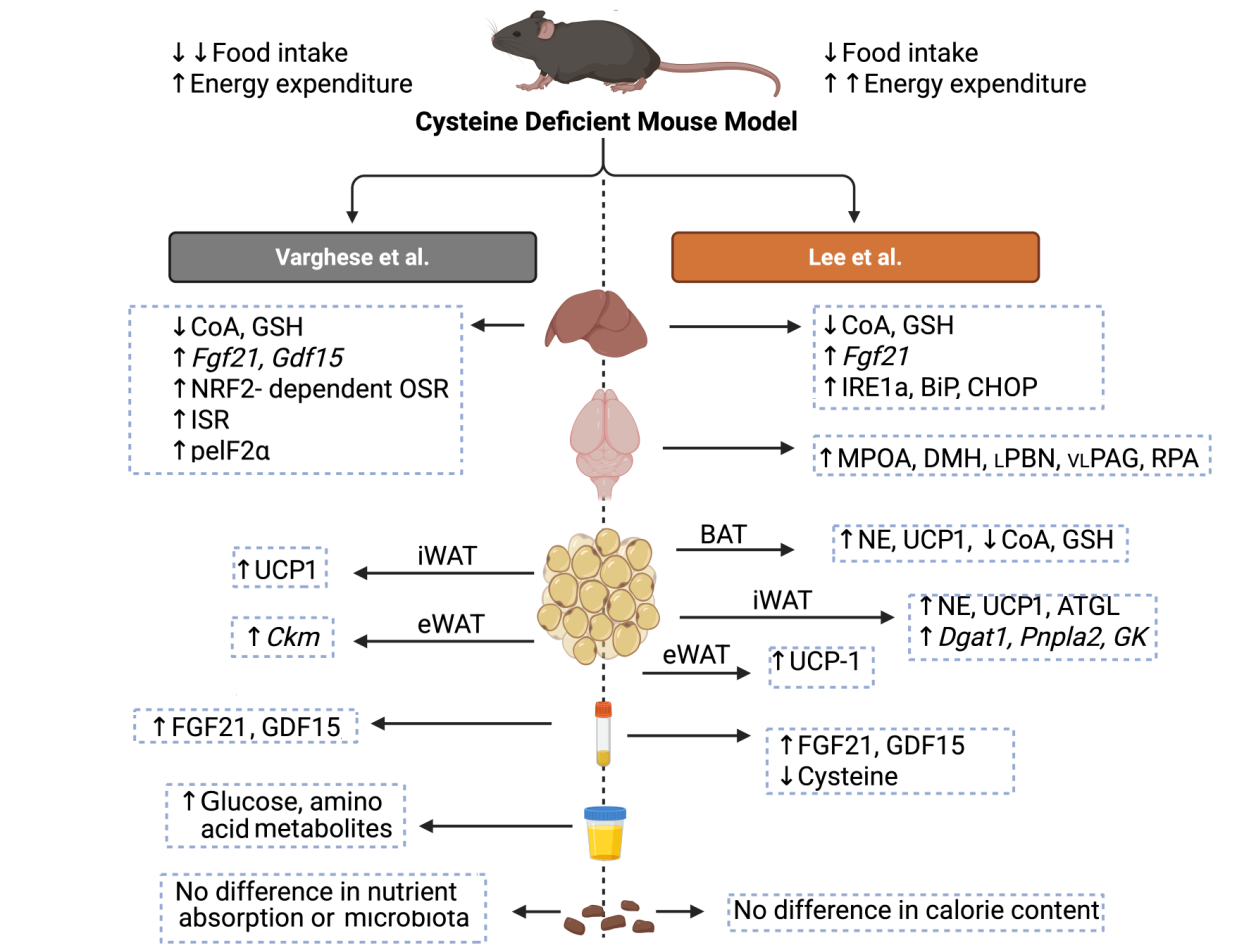
FIGURE 1: Summary of the main findings from the studies of Varghese et al 1 and Lee et al 2. Created in BioRender. Hankir, M. (2025) https://BioRender.com/1og4zh8.

With the rising prevalence of obesity, pharmacological weight loss treatments such as semaglutide have taken the world by storm. It is even predicted that bariatric surgery will increase in demand rather than the other way round as people living with obesity become more aware of the multitude of benefits associated with weight loss. The groundbreaking studies of Varghese et al 1 and Lee et al 2 provide compelling arguments that refocusing on nutrition can have equally effective results. Additionally, the reversibility of weight loss from cysteine depletion sets it apart from bariatric surgery. However, for the findings to be translated several major hurdles need to be overcome. Even if selective CSE/CTH inhibitors/gene therapies can be developed, the safety of cysteine depletion needs to be carefully evaluated in humans with a particular focus on duration 31. Nevertheless, because of the rapidity and magnitude of weight loss caused by cysteine depletion, it may have applications for acute measures rather than serving as a chronic treatment. For example, a very low-calorie diet is typically implemented for several weeks prior to bariatric surgery to reduce adiposity and liver size. Calibrated cysteine depletion in these patients leading to safe levels of weight loss may provide a more practical and efficient substitute. Several other questions and issues remain of course. Specifically, why does cysteine depletion have such a profound effect on adipose tissue? One potential explanation is that it causes a pseudo state of extreme starvation sensed by the liver that necessitates rapid mobilisation of energy stored as fat. In support of this idea, adipose triglyceride lipase (ATGL), the first enzyme involved in the breakdown of triglycerides, is strongly upregulated and activated in subcutaneous adipose tissue of CSH knockout mice deprived of cysteine 2. Thus, the marked browning of adipose tissue during cysteine depletion may simply be an epiphenomenon. Notably, while both Varghese et al 1 and Lee et al 2 found that housing CSH knockout mice deprived of cysteine at thermoneutrality had no metabolic effect, it would be interesting to determine if they are more tolerant to cold exposure. Another question that arises is the data of Varghese et al 1 suggests that weight loss from cysteine depletion is mainly driven by reduced food intake while the data of Lee et al 2 suggests it is mainly driven by increased energy expenditure (Figure 1). Along these lines, Lee et al 2 report strong activation of neurons in the lateral PBN during cysteine depletion in the context of adipose tissue thermogenesis. Considering the role of these neurons in promoting aversion to visceral toxins 32 and to semaglutide 33, it would be interesting to test their relevance for aversion in the context of cysteine depletion. This may make the intervention more prescribable since aversion is a major reason why patients discontinue semaglutide 34. Perhaps the most fascinating question that remains unanswered is the identity of the signal(s) released by hepatocytes that promote weight loss during cysteine depletion. Considering that half of this weight loss is not mediated by the sympathetic nervous system, this leaves open various forms of organ crosstalk mediated by novel hepatokines. Finally, the groundbreaking studies of Varghese et al 1 and Lee et al 2 attest to how merging fields as varied as biochemistry, molecular biology, cell biology, nutrition, endocrinology and neuroscience can unravel new signalling pathways and guide the development of alternative treatment options for obesity.

## CONFLICT OF INTEREST

The authors declare no conflict of interest.
